# Pamphlets Received

**Published:** 1895-09

**Authors:** 


### Pamphlets Received.

---

The Treatment of Anal Fissure or Irritable Ulcer of the Rectum.—By Lewis H. Adler, Jr., M. D. Reprint from Medical News, Philadelphia.

The Operative Treatment of Fistula in Ano.—By Lewis H. Adler, Jr., M. D. From International Medical Magazine, Philadelphia.

A Case of Didelphic Uterus with Lateral Hæmatocolpos, etc.—By X. O. Werder, M. D., Pittsburg. From Journal American Medical Association.

Abdominal Section in Ectopic Gestation Where the Fœtus is Living and Viable, with Report of a Successful Case—By X. O. Werder, M. D., Pittsburg. From Transactions of the Association of Obstetricians and Gynæcologists, 1894.

The Dilator in Diseases of the Air Passages.—By Seth Scott Bishop, M. D., Chicago. From National Popular Review.

Address on the Founding of the Illinois Hospital.—By S. S. Bishop, M. D., Chicago. From the Journal American Medical Association.

Treatment of Laryngeal Tuberculosis.—By Robert Levy, M. D., Denver. From New York Medical Journal, July 20th, 1895.

Imperforation of the Rectum.—By George Ben Johnston, M. D., Richmond, Va.

On Movable Kidney.—By George Ben Johnston, M. D., Richmond, Va. From Transactions Southern Surgical and Gynæcological Association, 1895.

Extirpation and Colotomy in Cases of Carcinoma of the Rectum.—By L. H. Adler, Jr., M. D., Philadelphia. From Medical News.

The Treatment of Fistulæ in Ano by Lange's Method of Immediate Suture of the Tract.—By L. H. Adler, Jr., M. D., Philadelphia. From Medical News.

Favorable Results of Koch's Tuberculin Treatment in Tubercular Affections that are Not. Pulmonary.—By Charles Dennison, A. M., M. D., Denver. From New York Medical Journal.

Antiphthisine.—By Charles Dennison, A.M., M.D., Denver. From Medical Record, New York.

The Treatment of Chronic Endometritis.—By X. O. Werder, M. D., Pittsburg. From Pittsburg Medical Review.

A Report of the Abdominal Sections in the Gynæcological Department of Mercy Hospital. —By Hubert A. Royster, M. D. Service of X. O. Werder, M. D. From Pittsburg Medical Review.

The Infiltration Method of Local Anæsthesia in Genito-Urinary Surgery.—By Bransford Lewis, M. D.

Medical Terminology, Its Etymology and Errors.—By P. J. McCourt, M. D., New York. From Medical Record.

A Case of Otitic Abscess in a Diabetic.—By W. Cheatham, A. B., M. D., Louisville. From Cincinnati Lancet-Clinic.

The Liver as an Organ of Elimination of Corpuscular Elements.—By Gustav Futterer, M. D. From Medicine, August, 1895.

A Case of Puerperal Pelvic Abscess, with Some Remarks on Septic Infection.—By George M. Boyd, M. D., Philadelphia. From American Gynæcological and Obstetrical Journal.

Two Cases of Tubal Pregnancy Operated Upon More Than a Month After Rupture. *Ibid.*

The Accouchement Force. *Ibid.*

Cystic Tumors of the Vaginal Vault, with Report of Two Cases.—By Frederick Holme Wiggin, M D., New York. From New York Medical Journal.

Strabismus as a Symptom, Its Causes and its Practical Management.—By Leartus Connor, M.D., Detroit. From Journal American Medical Association.

Cirrhosis of the Liver in Childhood.—By W. A. Edwards, M. D., San Diego, Cal. From Archives of Pædiatrics.

A Practical Low-Priced Device to Secure the Trendelenburg Posture.—By W. A. Edwards, M. D. From University Medical Magazine, Philadelphia.

Bicycling for Women from the Standpoint of the Gynæcologist.—By R. L. Dickinson, M. D. From American Journal of Obstetrics.

A Second Attack of Papillitis Occurring in a Case of Post-Neuritic Atrophy of the Optic Nerves.—By G. E. de Schweinitz, M.D., and A. G. Thomson, M. D., Philadelphia. From Archives of Ophthalmology.

Pre-Columbian Leprosy.—By Albert S. Ashmead, New York. From Journal American Medical Association.

Electrolysis of Small Growths, with Photographs and Report of a Case.—H. C. Bennett, M., D., Lima, Ohio. From St. Louis Courier of Medicine, May and June, 1895.

Puerperal Eclampsia.—By A. D. Price, M. D., Harrodsburg, Ky.

